# Lysosome (Dys)function in Atherosclerosis—A Big Weight on the Shoulders of a Small Organelle

**DOI:** 10.3389/fcell.2021.658995

**Published:** 2021-03-29

**Authors:** André R. A. Marques, Cristiano Ramos, Gisela Machado-Oliveira, Otília V. Vieira

**Affiliations:** iNOVA4Health, Chronic Diseases Research Center (CEDOC), NOVA Medical School (NMS), Universidade NOVA de Lisboa, Lisbon, Portugal

**Keywords:** lysosome dysfunction, atherosclerosis, oxidized lipids, autophagy, lysosomal storage diseases

## Abstract

Atherosclerosis is a progressive insidious chronic disease that underlies most of the cardiovascular pathologies, including myocardial infarction and ischemic stroke. The malfunctioning of the lysosomal compartment has a central role in the etiology and pathogenesis of atherosclerosis. Lysosomes are the degradative organelles of mammalian cells and process endogenous and exogenous substrates in a very efficient manner. Dysfunction of these organelles and consequent inefficient degradation of modified low-density lipoproteins (LDL) and apoptotic cells in atherosclerotic lesions have, therefore, numerous deleterious consequences for cellular homeostasis and disease progression. Lysosome dysfunction has been mostly studied in the context of the inherited lysosomal storage disorders (LSDs). However, over the last years it has become increasingly evident that the consequences of this phenomenon are more far-reaching, also influencing the progression of multiple acquired human pathologies, such as neurodegenerative diseases, cancer, and cardiovascular diseases (CVDs). During the formation of atherosclerotic plaques, the lysosomal compartment of the various cells constituting the arterial wall is under severe stress, due to the tremendous amounts of lipoproteins being processed by these cells. The uncontrolled uptake of modified lipoproteins by arterial phagocytic cells, namely macrophages and vascular smooth muscle cells (VSMCs), is the initial step that triggers the pathogenic cascade culminating in the formation of atheroma. These cells become pathogenic “foam cells,” which are characterized by dysfunctional lipid-laden lysosomes. Here, we summarize the current knowledge regarding the origin and impact of the malfunctioning of the lysosomal compartment in plaque cells. We further analyze how the field of LSD research may contribute with some insights to the study of CVDs, particularly how therapeutic approaches that target the lysosomes in LSDs could be applied to hamper atherosclerosis progression and associated mortality.

## Introduction

### Cardiovascular Diseases and Atherosclerosis

Cardiovascular diseases (CVDs) are a group of disorders affecting the function of the heart and blood vessels and constitute the current leading cause of death worldwide, claiming more lives than all forms of cancer combined (Virani et al., 2020). Atherosclerosis is the main contributor to this burden, being at the basis of various forms of CVD, including peripheral arterial disease, myocardial infarction and stroke (Mendis et al., 2011). The term atherosclerosis derives from the Greek “athere” (gruel) and “skleros” (hard), referring to the “waxy” plaques inside blood vessels described over 100 years ago. This disease is known to develop silently over decades without noticeable symptoms, until the patients experience harmful complications. Risk factors for atherosclerosis include hypertension, hypercholesterolemia, diabetes mellitus, obesity, and smoking (Libby et al., 2019). The factors and mechanisms that initiate atherogenesis are particularly complex and still not completely understood. Uncovering the molecular mechanisms driving plaque development in arterial cells is of the uttermost importance for the future development of more accurate disease biomarkers and more effective therapies.

### Major Steps of Plaque Development

#### Diffuse Intimal Thickening (DIT)

**FIGURE 1:**
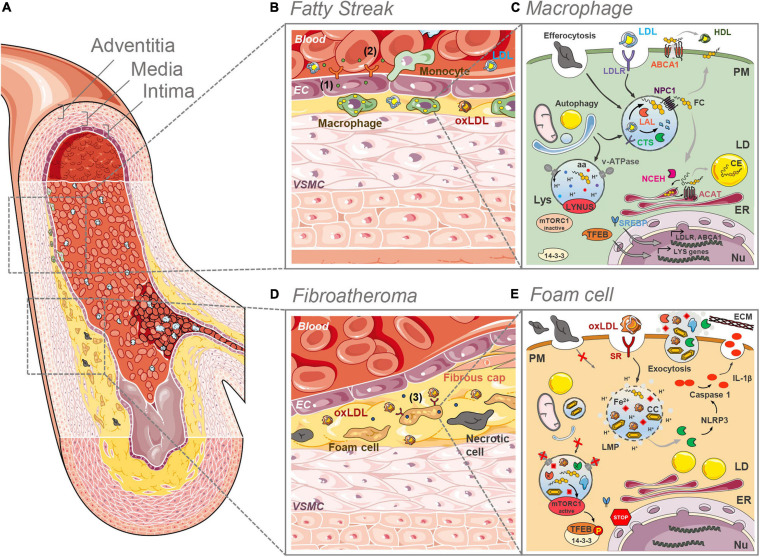
Lysosome (dys)funtion in the initial and final stages of plaque development. **(A)** Anatomically, large arteries consist of three well defined and morphologically distinct layers. The intima, the innermost layer in direct contact with the blood stream, comprising an endothelial layer surrounded by a connective tissue basement membrane with elastic fibers. The media is the thickest layer and consists primarily of VSMCs. The outermost adventitia, with connective tissue and varying amounts of collagenous and elastic fibers, attaches the artery to the surrounding tissue. **(B)** In the initial stages of plaque development (fatty streak) LDL infiltrates the intima, where it undergoes oxidation and other modifications. The formed oxLDL causes the proliferation of VSMCs and triggers the secretion of adhesion molecules (1) and chemoattractants (2) by ECs, which in turn recruit monocytes from the blood stream. In the intima, monocytes differentiate into macrophages that clear LDL and oxLDL. **(C)** In macrophages of the intima the LDL is taken up by the LDLR and routed to the lysosomes (Lys) where the acid lipase LAL hydrolases the CE and TG to FC and fatty acids. The cathepsin (CTS) proteases breakdown the protein apoB. FC is exported from the lysosomes by NPC1 and then directed to the different organelles (ER, PM, etc.). The ER enzyme ACAT esterifies cholesterol to CE, which are stored in cytosolic LDs. CE can be converted back to FC by NCEH. Excess FC is effluxed via the ABCA1 plasma membrane (PM) transporter. Efferocytosis and selective autophagy converge to the lysosomal compartment for degradation of substrates sequestered in phagosomes and autophagosomes, respectively. Intralysosomal hydrolysis dependents on the activity of the proton (H^+^) pump v-ATPase that maintains the intraluminal acidic pH. Nutrients such as aminoacids (aa) and FC are sensed by the LYNUS machinery of which mTORC1 is a part of. Abundance of nutrients dictates the inactivity of mTORC1 and the translocation of (14-3-3) free TFEB to the nucleus (Nu) to drive the transcription of lysosome and autophagy genes. The uptake of LDL is regulated by the action of the SREBP transcription factors. **(D)** In advanced plaques (fibroatheroma stage) a necrotic core is formed with lipid-laden foam cells and necrotic cells. Plaque rupture is prevented by the fibrous cap formed by VSMCs that migrated from the intima. Foam cells secrete pro-inflammatory cytokines aggravating local inflammation (3). **(E)** As a result of the unregulated uptake of oxLDL by SRs at the surface of foam cells, the lysosomal compartment becomes overloaded with partially digested oxLDL, FC and eventually CCs. The accumulation of these materials causes lysosomal membrane permeability (LMP), the loss of the proton gradient and the release of CTS into the cytosol. There, these proteases participate in the NRLP3 inflammasome activation cascade that culminates with the processing of pro-IL-1β into IL-1β by active caspase 1. The lysosomal iron (Fe^2+^) pool may be one of the factors determining a decrease in the activity of the v-ATPase besides contributing to further LDL oxidation. The elevated lysosomal pH reduces the activity of the hydrolases. The decreased lysosomal hydrolytic function causes an impairment in the efferocytic and autophagic capacity of the cell. The accumulation of FC may be the culprit for the hyperactivation of mTORC1 which phosphorylates TFEB causing its retention (in a complex with 14-3-3) in the cytosol. This prevents lysosome biogenesis. Exocytosis of lysosomal contents might contribute to atherogenesis through the release to the extracellular space of partially degraded materials and the release of CTS, whose activity contributes to LDL aggregation and ECM breakdown. Some images in this figure were adapted with permission from Servier Medical Art, licensed under a Creative Common Attribution 3.0 Generic License (http://smart.servier.com/).

Atherosclerotic lesions are predominantly located in the intima layer of middle-sized and large elastic and muscular arteries, especially at branch points and in areas of high vessel curvature (Lusis, 2000; Tabas and Bornfeldt, 2016; Figure 1A). These areas are subjected to low shear stress and disturbed flow, which modulate the expression and structure of permeability-related intercellular junctional proteins in endothelial cells (ECs) (Milutinović et al., 2020). As a consequence, the endothelial barrier becomes permeable, leading to the accumulation and sequestration of low-density lipoproteins (LDLs) in the intima (Colin et al., 2014). In response to pro-atherogenic stimuli, vascular smooth muscle cells (VSMCs) start to proliferate and to produce a high amount of modified extracellular matrix (ECM). This process underlies the occurrence of diffuse intimal thickening (DIT), also known as “fatty streak,” already present in human arteries even before atherosclerosis develops (Nakashima et al., 2008; Figure 1B).

#### Pathological Intimal Thickening (PIT)

In the early stages of atherosclerosis intima thickening becomes pathological (PIT), as the ECM facilitates the binding, retention, and deposition of further lipoprotein-derived lipids. The LDLs are subsequently modified by numerous mechanisms including oxidation and enzymatic cleavage, transforming them into pro-inflammatory oxidized LDL (oxLDL). These modified lipoproteins damage the endothelium, triggering a pro-inflammatory cascade initiated by the activation of ECs, which start secreting chemo-attractants [e.g., monocyte chemotactic protein-1 (MCP-1)] (Cushing et al., 1990) and adhesion molecules (e.g., ICAM-1, P-selectin, E-selectin, PCAM-1, and VCAM-1) (Davies et al., 1993; Tabas et al., 2007). The presence of these molecules causes local recruitment of leukocytes (mainly monocytes) from the bloodstream, which adhere and migrate into the vessel wall (Figure 1B). In the intima, monocytes differentiate predominantly into pro-inflammatory macrophages (Ross, 1999), which express the scavenger receptors (SRs) and internalize modified lipoproteins. The infiltration and proliferation of macrophages is a characteristic feature of the progression of PIT to fibroatheroma stage.

#### Fibroatheroma Stage

The pro-inflammatory plaque macrophages give rise to local inflammation by secreting pro-inflammatory cytokines [e.g., tumor necrosis factor alpha (TNFα), interleukin-6 (IL-6), and IL-1β] (Figure 1D), which recruit T and B cells to the lesion site (Zhou and Hansson, 1999). Akin to macrophages, VSMCs are efficient phagocytic cells that take up modified and oxLDL via SRs (as well as by micropinocytosis). As a consequence, VSMCs differentiate into macrophage-like cells (Chellan et al., 2018). This unrestrained uptake of oxLDL by macrophages and macrophage-like cells eventually takes a toll on the lysosomal compartment, responsible for the processing of the incoming lipoproteins. The saturation of the degradative capacity of lysosomes causes the accumulation of unprocessed lipids in these organelles and in cytosolic lipid droplets (LDs). These lipid-laden cells transform into the so-called foam cells, major players in the ensuing steps of the atherogenic cascade (Allahverdian et al., 2014; Vengrenyuk et al., 2015). Foam cells undergo apoptosis and accumulate calcium in the acidic extracellular space. Given that the now dysfunctional macrophages and VSMCs are the main cell types responsible for the efferocytic removal of dead cells and apoptotic bodies, a lipid-rich necrotic core starts to form in fibroatheroma (Libby et al., 2019; Figure 1D).

### Plaque Rupture and Thrombosis

Initially, the necrotic core is surrounded by a thick fibrous cap composed of VSMCs, which protects the vulnerable plaque (Durham et al., 2018). However, the unresolved inflammation leads to the production of cytokines that induce apoptosis of VSMCs or differentiation into cells with osteochondrogenic phenotype, which promote mineral deposition in the atherosclerotic plaque (Ceneri et al., 2017; Durham et al., 2018). Eventually, the continuous erosion of the fibrous cap, due to VSMC death and the degradation of collagen by metalloproteases, destabilizes atherosclerotic plaques. The final trigger to this pathogenic cascade is the expansion of the necrotic core due to intraplaque neovascularization and the extravasation of platelets and erythrocytes (Milutinović et al., 2020). The now thin-cap fibroatheroma is unstable and prone to rupture and thrombosis. Thrombotic events can then lead to myocardial infarction and stroke, with major clinical consequences such as death and disability.

### Lysosomal Dysfunction in Atherosclerosis

During plaque development, one organelle in particular endures a sheer amount of stress. Lysosomes, the recycling centers of the cell, must cope with the unrestrained amounts of lipoproteins taken up by foam cells, process the mounting apoptotic bodies engulfed by phagocytes and, as a nutrient sensing platform, regulate the unbalanced cellular metabolic status and growth rate. The giant task of these small organelles in atherosclerotic plaques was first recognized by the pioneering work of Christian De Duve (Peters and de Duve, 1974; Shio et al., 1974), Nobel prize recipient for the discovery of these acidic organelles (De Duve et al., 1955). De Duve and his team were the first to describe that the arterial cells of rabbits fed a cholesterol-rich diet progressively transform into foam cells due to a dysfunctional lysosomal compartment, unable to dispose of lipoproteins (Peters and de Duve, 1974; Shio et al., 1974). Various posterior studies have confirmed these ground-breaking observations (Goldfischer et al., 1975; Haley et al., 1977; Fowler et al., 1980; Bobryshev et al., 2013). In this review, we explore the most recent discoveries regarding the molecular details commanding the lysosomal dysfunction observed in plaque cells and the way in which this contributes to the ensuing pathogenic cascade.

## Atherosclerosis, an Acquired Lysosomal Storage Disease?

### Lysosomes

These small cytoplasmic organelles are spherical membrane-bound vesicles containing numerous acidic hydrolytic enzymes that can breakdown biological polymers, such as lipids, proteins, carbohydrates, and nucleic acids (Ballabio and Bonifacino, 2020). Lysosomes degrade and recycle extracellular material via endocytosis, macropinocytosis and phagocytosis, and intracellular material via autophagy. The catalytic function of lysosomes makes them indispensable for multiple cellular processes, including antigen presentation, pathogen inactivation, downregulation of surface receptors and turnover of cellular components (Saftig and Puertollano, 2020). Importantly, lysosomes not only recycle cellular building blocks, but rather constitute a key signaling hub, able to sense and integrate information about changing environmental conditions and quickly translate it into cellular adaptation (Saftig and Puertollano, 2020). The cellular metabolic and signaling status is relayed via the microphthalmia family of transcription factors (MiT/TFE) and determines the particular rate of lysosomal biogenesis, autophagy and exocytosis (Marques and Saftig, 2019).

To this date, four members of the MiT/TFE family have been identified: microphthalmia-associated transcription factor (MITF), transcription factor EB (TFEB), TFE3, and TFEC (Steingrímsson et al., 2004). All four members of the MiT/TFE family have the ability to bind a unique enhancer box (E-box) DNA motif [also referred to as CLEAR (coordinated lysosomal expression and regulation)] within the proximal promoters of lysosomal and autophagy genes (Sardiello et al., 2009). TFEB is considered the master regulator of lysosome biogenesis and therefore plays a central role in the control of cell and organismal homeostasis (Sardiello et al., 2009; Settembre et al., 2013; Napolitano and Ballabio, 2016). The subcellular localization and activity of TFEB are regulated by the mechanistic target of rapamycin (mTOR)-mediated phosphorylation, a kinase present at the lysosomal surface (Napolitano and Ballabio, 2016). Under nutrient−rich conditions, TFEB is phosphorylated by the mTOR complex 1 (mTORC1) kinase and retained in the cytoplasm bound to the 14-3-3 chaperone protein (Sancak et al., 2010). Other growth-regulating kinases such as MAPK kinase (MEK)/extracellular signal-regulated kinase (ERK) and glycogen synthase kinase 3 (GSK3) can also promote TFEB phosphorylation (Perera et al., 2019). In response to starvation and lysosomal stress, TFEB is dephosphorylated by the calcium-dependent phosphatase calcineurin, translocating to the nucleus and inducing the transcription of target genes (Settembre et al., 2011; Medina et al., 2015). Under oxidative stress conditions TFEB and TFE3 can also be dephosphorylated by the action of protein phosphatase 2A (PP2A), causing their nuclear translocation (Martina and Puertollano, 2018). Defects that impair any of the functions of lysosomes, cause the accumulation of undigested or partially digested macromolecules in their interior (that is, “storage”) or impair the transport of molecules, which can result in cellular damage.

### Lysosomal Storage Disorders

Lysosomes are comprised by more than 70 hydrolases, 200 membrane proteins and numerous other associated proteins (Saftig and Puertollano, 2020). Of the ∼1,300 genes involved in lysosomal function, approximately 70 monogenic disorders of lysosomal catabolism have been described. These are classified as Lysosomal Storage Diseases (LSDs), heritable (inborn) errors of metabolism affecting the function of the lysosome, most of which are inherited as autosomal recessive traits (Platt et al., 2018). These disorders are individually rare, but collectively affect 1 in 5,000 live births (Platt et al., 2018). LSDs typically manifest in infancy and childhood, although adult-onset forms also occur. Most LSDs have a progressive neurodegenerative clinical course, yet symptoms in other organ systems are frequent. Cardiac manifestations are a common finding in many LSDs, underscoring the fundamental role of lysosomes in the homeostasis of the cardiovascular system (Laney et al., 2018; Chi et al., 2020).

### Cardiovascular Pathology in LSDs

The most severe cardiovascular manifestations in LSDs have prenatal onset, for example, cardiomyopathy in infantile-onset Pompe disease (lysosomal acid alpha-glucosidase deficiency), while other manifestations occur more subtly at later stages and become more life-threatening over time, in conditions such as Fabry disease (alpha-galactosidase A deficiency) (Laney et al., 2018). In the adult variants of LSDs, cardiovascular manifestations can be pathognomonic for the disease itself, but often occur earlier than expected (Laney et al., 2018). Patients suffering from an attenuated form of lysosomal acid lipase (LAL) deficiency, also known as cholesterol ester storage disease (CESD), provide a prototypical example. CESD patients suffer from premature atherosclerosis and early onset CVD due to unfavorable lipid profiles in adolescence that may be aggravated by other risk factors such as elevated body mass index (BMI) (Erwin, 2017). Cystinosis patients, with mutations in the gene encoding for the lysosomal cysteine transporter (CTNS), are also at high risk for the development of vascular calcifications and obstructive atherosclerosis (Ueda et al., 2006). Further highlighting the role of the autophagy-lysosomal system in cardiovascular homeostasis, deficiency in the lysosome-associated membrane protein 2 (LAMP2) (one of the main lysosomal membrane glycoproteins) causes Danon disease, a pathology characterized by massive autophagosome deposition in cardiomyocytes (Platt et al., 2018; Zhu et al., 2020). LAMP2 deficiency leads to autophagy impairment and autophagosome aggregation in these cells, resulting in cardiac defects (Tanaka et al., 2000). Even single defects in the lysosome machinery, such as represented by monogenic LSDs, can thus have a significant impact on the cardiovascular system.

### Acquired LSDs

The concept of LSD has been broadened to encompass acquired pathologies in which malfunction of the autophagy-lysosomal system plays a significant etiological role (Marques and Saftig, 2019). External and acquired factors, such as diet or pathogen infection, can impact the function of lysosomes. The term “acquired LSDs” has thus been coined to refer to more common human pathologies presenting a malfunctioning lysosomal compartment, including neurodegenerative diseases, kidney and cardiovascular diseases, as well as other age-related diseases. Christian de Duve himself defined foam cell formation in atherosclerosis as a variant of LSD (Peters and de Duve, 1974; Shio et al., 1974; Jerome, 2006). Therefore we will now turn our attention to the molecular mechanisms that have attested to the hypothesis since it was raised by De Duve almost 50 years ago.

## The Role of Lysosomes in Foam Cell Formation

### Lysosomal LDL Processing

In early atherosclerotic lesions, monocyte-derived macrophages take up the retained lipoproteins through receptor-mediated endocytosis and fluid-phase pinocytosis (Figure 1C). Native LDL are mainly recognized by the LDL receptor (LDLR), which is responsible for the cellular endocytosis process (Brown and Goldstein, 1986). The uptake of LDL particles is a highly regulated system with regulatory feedback mechanisms through suppression of the sterol-regulatory element-binding protein (SREBP) pathway to avoid excessive uptake and lipid overload (Brown and Goldstein, 1997; Figure 1C). The endocytosed LDL are shuttled to lysosomes in order to be metabolized. During that process, cholesteryl esters (CE) are hydrolyzed by the LAL into free cholesterol (FC) and fatty acids (Ouimet et al., 2011; Sergin and Razani, 2014). FC is rapidly exported out of the lysosome by the joint action of the Niemann-Pick C1 and C2 (NPC1 and NPC2) proteins (Figure 1C). Membrane contact sites mediate cholesterol delivery to other organelles, including the endoplasmic reticulum (ER) and peroxisomes (Meng et al., 2020). The excess of FC can be effluxed to the periphery through ATP-binding cassette transporter A1 (ABCA1)-mediated efflux to nascent high-density lipoproteins (HDL) (Brown et al., 1980; Figure 1C). However, if the influx of LDL-derived cholesterol is higher than the efflux, the excess of FC is re-esterified in the ER by acyl-CoA:cholesteryl acyltransferase (ACAT) and stored in the cytosol in LDs in the form of CE (Meiner et al., 1996; Figure 1C). Cholesterol re-esterification is a regulated and protective mechanism indispensable to prevent excessive FC accumulation in membranes, mainly in the ER, which could be toxic for the cell (Brown and Goldstein, 1983). Even though LD accumulation eventually leads to foam cell formation (Brown et al., 1980), this process is essentially reversible. When the rate of cholesterol efflux is sufficient, the action of the cytoplasmic enzyme neutral cholesterol-ester hydrolase (NCEH) can convert CE back to FC for use by the cell (Figure 1C). Alternatively, cells can generate FC through the autophagic uptake of LDs with subsequent LAL-dependent degradation of CE, a form of selective autophagy named lipophagy (Ouimet and Marcel, 2012). This cycle of hydrolysis and re-esterification is continuous and has a half-life of approximately 24 h (Gonen and Miller, 2020).

### LDL Oxidation

The LDL and other apoB-containing particles accumulated in the artery wall may undergo extracellular and intracellular (lysosomal) modifications, which are associated with increased atherogenicity. These cholesterol-containing structures may be oxidized, aggregated, or enzymatically modified. These modifications significantly affect the physicochemical and biological properties of the lipoproteins (Aviram, 1993; Hoff and Hoppe, 1995). One of the major chemical modifications of LDL is oxidation, involving free radical mediated lipid peroxidation, which was already demonstrated to occur *in vivo* (Gibson et al., 2018; Domingues et al., 2021). The presence of different subsets of lipases and oxygenases in the arterial wall may result in the occurrence of numerous forms of modified LDL *in vivo*. OxLDL is thus an umbrella term that describes a vast mixture of over 3,000 molecules, most of which with biological activity (Gibson et al., 2018). In the field of atherosclerosis a debate persists regarding the exact mechanisms by which LDLs are oxidized in the interior of arteries (see “*Lysosomal Iron Metabolism”* section). Regardless of their origin, these modifications decrease the ability of LDL to bind to the LDLR (Avogaro et al., 1991). The pro-atherogenic potential of oxLDL has been solidly established (Goldstein et al., 1979; Steinberg et al., 1989; Jerome and Lewis, 1990; Aviram, 1993), although the role of each particular bioactive molecule remains largely unexplored. While most research has focused on the atherogenicity of oxidized phospholipids and oxysterols, our recent studies suggest that the end products of CE oxidation, cholesteryl hemiesters, may be responsible for some of the pro-atherogenic properties ascribed to oxLDL (Estronca et al., 2012; Domingues et al., 2017, 2021).

### OxLDL-Induced Lysosomal Dysfunction

As the lesion progresses, foam cells arise as the result of the uncontrolled uptake and defective metabolization of modified-LDL accumulated in the vessel wall by cells. The uptake of modified lipids by phagocytes, namely macrophages and VSMCs, is mediated by alternate SRs (Figure 1E). These receptors include lectin-like receptors (LOX), SR-class A, CD36, and toll-like receptors (TLR) (Hoff and Hoppe, 1995; Kunjathoor et al., 2002; Bolick et al., 2009; Adamson and Leitinger, 2011), and in contrast to LDLR their expression is not down-regulated by increased intracellular cholesterol levels (Miller et al., 2005; Mehta et al., 2007; Choi et al., 2009; Manning-Tobin et al., 2009). The lack of a negative feedback regulation results in excessive intracellular storage of these lipids (Röhrl and Stangl, 2018). Furthermore, the uptake of oxLDL by SRs blocks the normal handling of cholesterol at the endolysosomal stage (Figure 1E). Studies indicate that only approximately 50% of oxLDL are effectively degraded in lysosomes, in part due to their resistance to the proteolytic activity of lysosomal proteases (Lougheed et al., 1991; Jessup et al., 1992; Yancey and Jerome, 1998). Defective oxLDL hydrolysis results in their trapping in the lysosomal compartment, decreasing the exit of FC from the lysosomes to be esterified by ACAT (Lougheed et al., 1991). In time this causes lysosomal engorgement, due to further CE accumulation, and ultimately cholesterol crystal (CC) formation (Sheedy et al., 2013; Figure 1E). The progressive lysosomal dysfunction in plaque foam cells constitutes a form of lipidosis, i.e., irreversible lipid accumulation, a condition very similar to the one observed in LSDs such as Wolman and Niemann-Pick type C (NPC) diseases (Marques and Saftig, 2019).

## Lysosomal Dysfunction in Foam Cells

A large number of open questions still surround the mechanisms by which oxLDL triggers lysosomal lipidosis. Nonetheless, considerable advances have been made in recent years concerning the characterization of the multiple processes driving the impairment of lysosomal homeostasis in atherosclerotic plaque cells. Numerous studies have demonstrated that oxLDL, CC, and other modified lipids are able to alkalize lysosomal pH, impair the degradative capacity of lysosomal hydrolases and cause damage to the lysosomal membrane (Cox et al., 2007; Jerome et al., 2008; Sheedy et al., 2013; Emanuel et al., 2014).

### Increased Lysosomal pH

Each cell contains different populations of lysosomes, which can be distinguished by their position, size, acidification and reformation properties (Bright et al., 2016). Hydrolysis of cargo takes place in the perinuclear lysosomal population, characterized by the acidic pH (4.5–5.0). The intralysosomal acidic pH is the result of an electrochemical gradient maintained mostly by the vacuolar type-ATPase (v-ATPase) (Figure 1C), aided by the chloride channel CLC-7 (Mindell, 2012). One of the first observations made in macrophages exposed to oxLDL, was the inability of these cells to maintain an acidic lysosomal pH (Cox et al., 2007; Emanuel et al., 2014). In these lipid-engorged cells, the intralysosomal accumulation of FC directly inhibits the activity of the v-ATPase at the lysosomal membrane (Cox et al., 2007; Jerome et al., 2008). Another way by which oxLDL and the accumulated CC may cause alkalization of the lysosomal pH is through the induction of damage to the lysosomal membrane, causing leakage of the intraluminal contents and consequent loss of the proton gradient (Li et al., 1998a, b; Yuan et al., 2000; Emanuel et al., 2014; Figure 1E). The increase in lysosomal pH appears to be one of the prime insults affecting this organelle and triggering the ensuing malfunction.

### Impaired Degradative Capacity

All lysosomal hydrolases, responsible for the catabolism of endogenous and exogenous substrates, have optimal activity at acidic pH. The raise in lysosomal pH observed in foam cells impairs the ability of these enzymes to break down the incoming modified lipoproteins (Brown et al., 2000). This drop in lysosomal hydrolytic capacity has been firmly established in various atherosclerosis models (Jerome and Lewis, 1990; Yancey and Jerome, 2001; Griffin et al., 2005). LAL is the primary enzyme responsible for the hydrolysis of CE derived from lipoproteins. Alkalization of lysosomal pH by oxLDL was shown to directly impair LAL-mediated CE hydrolysis (Cox et al., 2007). Another class of enzymes fundamental for the catabolism of lipoproteins are cathepsin proteases, that catalyze the hydrolysis of apoB proteins. Several studies have shown that loading of cells with oxLDL increases the resistance of proteins to being degraded by cathepsins, namely cathepsin D (the most abundant lysosomal protease) and cathepsin B (Lougheed et al., 1991; Jessup et al., 1992). The ability of lysosomal cathepsins to degrade apoB appears to be directly inhibited by the phospholipids in oxLDL (O’Neil et al., 2003), in the case of cathepsin D, or in the case of cathepsin B by the covalent binding of the lipoproteins’ aldehydes to the cysteine and histidine residues on the enzyme’s active site (Hoppe et al., 1994; O’Neil et al., 1997; Crabb, 2002). This may, in turn, hamper the LAL-mediated hydrolysis of the CE in the core of oxLDL (Dubland and Francis, 2015). In accordance, histological studies of human atherosclerotic plaques have demonstrated the presence of lysosomes loaded with undegraded or partially degraded oxLDL (Mitchinson et al., 1985; Figure 1E). This inability to process oxLDL can eventually lead to the formation of toxic ceroid-lipofuscin aggregates in the foam cells of atherosclerotic lesions (Mitchinson, 1982; Mitchinson et al., 1985). The formation of these polymerized products of lipid oxidation is known to occur with aging and neurodegenerative disease, as well as in the neurological LSD neuronal ceroid lipofuscinosis (NCL), as a consequence of impaired lysosomal hydrolytic capacity (Seranova et al., 2017; Marques et al., 2019).

### Lysosomal Membrane Damage

OxLDL and CC, formed as a consequence of lysosomal FC accumulation, can cause damage to the lysosomal membrane. In macrophages, oxLDL- and CC-mediated lysosomal membrane permeabilization (LMP) causes loss of proton gradient and a massive release of lysosomal hydrolases into the cell cytosol (Figure 1E; Sheedy et al., 2013; Sergin et al., 2015). Downstream consequences of lysosomal leakiness may include NOD-like receptor family pyrin domain containing 3 (NLRP3) inflammasome activation (see “*Inflammation”* section) and cell death responses [for information on lysosomal-mediated cell death pathways see Aits and Jäättelä (2013)]. The mechanisms by which cells respond to LMP have only recently begun to be elucidated. Limited damage to the lysosomal membrane triggers the calcium-dependent recruitment of the ESCRT-III machinery to the place of the rupture and prompt repair (Skowyra et al., 2018). When the damage is too significant and repair is no longer an option, lysosomal proteins are selectively ubiquitinated, marking the damaged organelles to be sequestered by autophagosomes and eventually incorporated into autolysosomes for degradation (Hung et al., 2013; Maejima et al., 2013). This selective autophagic disposal of damaged lysosomes was termed lysophagy. As we will discuss below (see “*Autophagy”* section), autophagy is disturbed in atherosclerosis, and the removal of damaged lysosomes may thus be impaired, contributing to the aggravation of the pathogenic cascade.

## The Impact of Lysosomal Dysfunction in Atherogenesis

Lysosomes are indispensable players in several mechanisms that affect atherosclerotic disease progression, including inflammation, efferocytosis, exocytosis, autophagy, mTOR signaling, and iron metabolism. The irreversible lipid accumulation taking place in the lumen of lysosomes of advanced plaque cells severely affects the ability of the autophagy-lysosomal axis to perform these fundamental functions, further aggravating the pathological cascade through, for example: iron-mediated lysosomal oxidation of lipoproteins, exocytosis of partially degraded oxLDL, inefficient disposal of apoptotic bodies and inflammasome activation. Next, we will discuss the most relevant consequences of lysosomal dysfunction for plaque development.

### Lysosomal Iron Metabolism

It is consensual that atherogenesis begins with the oxidation of LDL in the vascular wall, yet the molecular details of this process are still the subject of debate (Steinbrecher et al., 1984; Leake and Rankin, 1990; Steinberg, 2009). It is known that lipid peroxidation requires the presence of transition metals such as copper and iron. Both metals, due to their redox potential, can catalyze the oxidation of polyunsaturated fatty acid moieties in LDL particles. Atherosclerotic plaques present elevated levels of iron and copper (Stadler et al., 2004), however, these metals exist in tightly bound forms and are therefore not readily available in the plasma and interstitial fluid (Dabbagh and Frei, 1995; Ahmad and Leake, 2019; Wen et al., 2019). Lysosomes are crucial for the regulation of the metabolism/homeostasis of iron and copper (Polishchuk and Polishchuk, 2016; Kim et al., 2021). In fact, the biggest pool of redox-active iron within cells is present in these organelles (Petrat et al., 2001; Wen and Leake, 2007). Lysosomal iron is thus a candidate for catalyzing the oxidation of LDL *in vivo*. Indeed, the lysosomal acidic environment increases the solubility of iron facilitating oxidation (Satchell and Leake, 2012). Furthermore, it has been demonstrated that LDL can be oxidized within the lysosomes of macrophages (Lamb and Leake, 1994; Ojo and Leake, 2018; Ahmad and Leake, 2019). In contrast, when LDL are incubated with copper at acidic pH its oxidation is delayed (Morgan and Leake, 1995), indicating that LDL oxidation by copper within lysosomes is unlikely to occur. Nonetheless, copper was shown to play an important role in regulating the lipolysis of triglycerides (TG) via cAMP signaling (Heffern et al., 2016). Therefore copper deficiency results in elevated hepatic TG levels (Xiao et al., 2018), which have been linked to atherosclerosis.

In recent years a novel link between lysosomal acidity and iron availability has been established (Yambire et al., 2019; Weber et al., 2020). Iron is delivered to lysosomes via receptor-mediated endocytosis of transferrin (Tf) or autophagocytosis of ferritin and organelles such as mitochondria (via mitophagy, the selective elimination of non-functional and damaged mitochondria) (Gammella et al., 2017). The ferritin and metalloproteins thus delivered to lysosomes are degraded to form low molecular weight iron (Kurz et al., 2007; Lv and Shang, 2018). Iron-loaded Tf is endocytosed by binding the transferrin-receptor (TfR1). As a consequence of vesicle acidification, iron is released from Tf and transported to the cytoplasm, while Tf and TfR1 are recycled back to the cell surface (Ullrich et al., 1996). Once iron dissociates from Tf, the ferric iron must be reduced by STEAP proteins before it is transported out of the endocytic compartment by the Divalent metal transporter 1 (DMT1) (Touret et al., 2003). Inhibition of the lysosomal v-ATPase impairs iron metabolism, ultimately leading to loss of mitochondrial function and cell death (Yambire et al., 2019). Additionally, iron uptake was shown to be essential to maintain cell proliferation when lysosomal pH is altered (Weber et al., 2020). The v-ATPase-iron-mitochondria axis is thus fundamental for cellular homeostasis and proliferation. Future research should elucidate whether this axis plays a role in atherogenesis, given the noted altered lysosomal pH and iron metabolism in atherosclerotic plaques. In this regard, elevated levels of ferritin in atherosclerotic arteries have been associated with myocardial infarction (Salonen et al., 1992; You et al., 2003), while Tf levels show a negative correlation with coronary heart disease by binding labile free iron (Das De et al., 2015).

### Inflammation

Lysosomes play a decisive role in cytokine release during atherosclerosis progression (Gibson et al., 2018; Hassanpour et al., 2019). Particularly, secretory lysosomes facilitate the release and the degradation of cytokines that require non-conventional secretion (Gibson et al., 2018). This “secretory” lysosome subset can either promote or suppress inflammation, depending on the stage of the inflammatory response. Another important aspect to consider is the role played by lysosomes in the activation of the NLRP3 inflammasome. NLRP3 inflammasome activation results in the maturation and release of IL-1β, a cytokine with a fundamental role in establishing and driving the pathogenesis of atherosclerosis (Gibson et al., 2018). NLRP3 activation in macrophages requires two signals. The first “priming” signal is NLRP3 transcription through NF-κB or breast cancer 1 (BRCA1)–BRCA2-containing complex (BRCC) 3 activation, leading to the synthesis of biologically inactive pro-IL-1β. The second signal can be delivered by a number of different sources, including dysfunctional lysosomes (Tall and Yvan-Charvet, 2015). This signal consists of NLRP3 activation and apoptosis-associated speck-like protein containing CARD (ASC) phosphorylation, which leads to the formation of the cytosolic NLRP3 inflammasome complex. This multiprotein complex is responsible for the synthesis of a mature form of caspase-1, which in turn cleaves pro-IL-1β, producing the mature and bioactive cytokine (Figure 1E). Regarding IL-1β release, several mechanisms have been described, including exocytosis by secretory lysosomes, exosomes, and microvesicle shedding (Gibson et al., 2018). As mentioned before, cholesterol oxidation products and CC affect the permeability of the lysosomal membrane, causing the leakage of hydrolases into the cytosol (Li et al., 1998a; Yuan et al., 2000; Emanuel et al., 2014). The release of cysteine proteases cathepsin B and L from these dysfunctional lysosomes can, at least partially, provide the second signal necessary for NLRP3 activation (Duewell et al., 2010). Both oxLDL and exogenous CC can induce IL-1β release in macrophages, even in the absence of other stimuli, demonstrating they provide both signals required for NLRP3 activation (Duewell et al., 2010; Sheedy et al., 2013). Additionally, NLRP3 inflammasome activation by lysosomal destabilization also requires particulate matter-mediated K^+^ efflux, although the exact mechanism linking these two events is unclear (Muñoz-Planillo et al., 2013; He et al., 2016). The molecular details of NLRP3 activation fall outside the scope of this review and have been discussed elsewhere (Karasawa and Takahashi, 2017; Gibson et al., 2018; Jin and Fu, 2019).

### mTOR Signaling

mTOR is a constitutively active kinase found in two different multiprotein complexes, mTORC1 and mTORC2 kinase complexes. Lysosomes receive information from the extracellular environment regarding the nutritional status of the cell via a complex machinery referred to as lysosomal nutrient sensing (LYNUS) (Napolitano and Ballabio, 2016). The LYNUS machinery is present at the lysosomal surface and generates a signaling response through mTORC1 (Zoncu et al., 2011; Bar-Peled and Sabatini, 2012). Cholesterol is among the nutrients sensed at the lysosomal membrane, regulating cellular signaling as well as cell proliferation and autophagy via the activation of mTORC1 (Castellano et al., 2017; Meng et al., 2020; Figure 1C). In NPC disease (NPC1 and NPC2 deficiency), intralysosomal FC accumulation due to defective export causes mTORC1 hyperactivation, mitochondrial dysfunction and impaired mitophagy, underlying the pathognomonic neurodegeneration (Davis et al., 2021). Given that mitophagy is a protective mechanism against oxLDL-mediated apoptosis of VSMCs (Swiader et al., 2016; Docherty et al., 2018; Yang Y. et al., 2020), safeguarding plaque stability, it would be of interest to explore the contribution of aberrant cholesterol-mTORC1 signaling to organelle pathogenesis in atherosclerosis.

In the case of macrophages, the mTOR pathway is not only a key sensor of nutrient status, but also the coordinator of metabolic and inflammatory signals determining activation of these cells (Vergadi et al., 2017). Macrophages can thus obtain an array of activation phenotypes, depending on the signals. These stages of macrophage activation can be broadly described as M1 and M2 polarization, depending on the activation stimulus. The metabolic and inflammatory signals converge to the PI3K/Akt/mTOR signaling pathway, that regulates the response of macrophages by modulating their activation phenotype (Vergadi et al., 2017). In particular, mTORC1 hyperactivation impairs the ability of macrophages to respond appropriately to polarizing stimuli by downregulating the downstream activity of Akt (protein kinase B) (Byles et al., 2013). In agreement, Razani and colleagues report that mTORC1-deficient mice present reduced atherosclerosis and plaque complexity (Zhang X. et al., 2019). They, however, suggest a divergent role for mTORC2 signaling in atherosclerosis, seeing as macrophage-specific mTORC2-deficient mice present larger and more complex lesions (Zhang X. et al., 2019). These differences may be explained by distinct impacts in macrophage polarization. Signaling by mTORC2 appears to inhibit the FoXO1 transcription factor, causing a suppression of the pro-inflammatory phenotype, particularly inflammasome activation and IL-1β levels (Su et al., 2009; Zhang X. et al., 2019). Curiously, they also report that deletion of macrophage mTOR, ablating both mTOR-dependent pathways, leads to residual alterations in atherosclerosis, reflecting the opposing effects of these two signaling pathways (Zhang X. et al., 2019).

### Exocytosis

Conventional lysosomes are not secretory organelles. Nonetheless there is strong evidence supporting the existence of an “unconventional” secretory pathway involving lysosomes, known as lysosomal exocytosis (Ballabio, 2016). This process implies the secretion of lysosomal contents upon fusion with the plasma membrane, an important mechanism of cellular clearance, necessary to maintain cell fitness (Buratta et al., 2020). This “unconventional” secretion involves a small fraction of lysosomes localized in the vicinity of the plasma membrane. In the first step of lysosomal exocytosis, the peripheral lysosomes are transported to the close proximity of the cell surface via microtubules, kinesin motors and small G proteins in a Ca^2+^-independent manner (Jaiswal et al., 2002; Encarnação et al., 2016). The docking and fusion of peripheral lysosomes with the plasma membrane is controlled by TFEB, in a process that requires the lysosomal calcium channel TRPML1 (transient receptor potential mucolipin 1) (Medina et al., 2011) and the calcium-sensor synaptotagmin (Reddy et al., 2001). Several lines of evidence suggest that increased lysosomal exocytosis may exacerbate atherogenesis by releasing to the extracellular space undigested material that is later engulfed by phagocyting macrophages (Gibson et al., 2018; Figure 1E). Supporting this claim, increased levels of multiple lysosomal hydrolases have been detected in atherosclerotic plaques (Hakala et al., 2003; Cheng et al., 2011). Among these enzymes with increased extracellular activity can be counted LAL, as well as the proteases cathepsin B, L, K, S, and D (Sukhova et al., 1998; Hakala et al., 2003; Liu et al., 2006a, b; Platt et al., 2007; Li et al., 2009; Cheng et al., 2011). Extracellular LAL and cathepsin D activity contribute to the hydrolytic modification of LDL, promoting its uptake by macrophages (and VSMCs) and subsequent foam cell formation (Hakala et al., 2003; Zhao and Herrington, 2016). Cathepsins B and S, on the other hand, have been shown to degrade the ECM and thereby increase plaque vulnerability (De Nooijer et al., 2009; Zhao and Herrington, 2016; Figure 1E). Increased levels of these hydrolases are not restricted to the arterial extracellular space, since elevated cathepsin B, S, and D levels have been detected in the circulation of patients suffering from CVDs. The circulation levels of the hydrolases appear to be related with increased risk of cardiovascular events (Naseem et al., 2005; Liu et al., 2006a; Wuopio et al., 2018).

### Autophagy

Autophagy is a highly evolutionarily conserved process with crucial roles in the degradation and recycling of long-lived or damaged intracellular material, including accumulated lipids (Martinet and De Meyer, 2009; Yang et al., 2018). Autophagy starts with the *de novo* formation of double-membrane–bound vesicles that elongate and sequester the cytosolic components, forming autophagosomes. These vesicles then fuse with hydrolase-rich lysosomes, forming autolysosomes, the vesicles in which proteolysis of substrates takes place. Nutrient deprivation triggers non-selective autophagy involving random uptake of cytoplasm portions, while selective autophagy specifically removes certain components, such as protein aggregates and damaged or superfluous organelles (Gatica et al., 2018). Cellular components targeted for selective autophagy are first recognized by “autophagy receptors,” which then interact with adaptor proteins that recruit the core autophagy-related (ATG) proteins, initiating autophagosome formation.

As mentioned above, the selective autophagic degradation of LDs—lipophagy—in plaque macrophages is crucial for maintaining cholesterol homeostasis in these cells, by delivering CE for lysosome-mediated cholesterol efflux (Singh et al., 2009). Several groups have reported an impairment of autophagy in plaque macrophages as a consequence of (modified) lipid-induced lysosomal stress and dysfunction (Yancey and Jerome, 1998; Duewell et al., 2010; Peden et al., 2011; Wild et al., 2011; Razani et al., 2012; Sheedy et al., 2013). Besides impaired lipophagy, other potential mechanisms underlying this observation may include hyperactivation of the inflammasome and IL-1β signaling, defective efferocytosis, and increased cell death due to accumulation of cytotoxic protein aggregates (Sergin et al., 2017; Evans et al., 2018). Additionally, a recent publication demonstrated that SR class B type I (SR-BI) is necessary to maintain macrophage autophagy in atherosclerosis through a mechanism involving TFEB expression and VPS34/Beclin-1 recruitment (Tao et al., 2021).

Researchers have corroborated autophagy’s vital role in preventing plaque formation, by demonstrating that disruption of this mechanism in mouse macrophages (by deletion of the essential autophagy protein ATG5) leads to marked increases in atherosclerosis (Ouimet et al., 2011; Liao et al., 2012; Razani et al., 2012). The group of Razani demonstrated that the acceleration of plaque formation under these conditions is due to an exacerbation of CC accumulation and subsequent hyperactivation of the NLRP3 inflammasome and selective IL-1β secretion (Sergin et al., 2017). Vindicating these studies, they have also reported a general decrease in autophagy in human and murine advanced atherosclerotic lesions (Sergin et al., 2017), featuring the presence of inclusion bodies with p62/SQSTM1, a chaperone for selective autophagy of cargo such as protein aggregates (Razani et al., 2012; Sergin et al., 2016). These inclusion bodies were shown to stem from plaque macrophages with impaired lysosomal degradative capacity, and consequently progressive autophagy dysfunction. Based on this cumulative evidence, the group proposes the pharmacological modulation of the autophagy-lysosomal biogenesis in macrophages as a promising therapeutic avenue for atherosclerosis (see the “*Boosting Lysosome Biogenesis”* section) (Sergin et al., 2017; Evans et al., 2018).

In ECs, oxLDL and other atherogenic insults stimulate autophagy to promote cell survival (Torisu et al., 2016). This pro-survival mechanism is countered, however, by low/disturbed shear stress, which impairs autophagy, promoting apoptosis and senescence (Vion et al., 2017). Additionally, this protective effect can also be attenuated by prolonged exposure to oxLDL, which eventually inhibits autophagy, especially lipophagy (Mollace et al., 2015; Zhang et al., 2020). Similarly, in VSMCs low concentrations of oxLDL or its isolated components can also induce autophagy (Ding et al., 2013; He et al., 2013), while higher concentrations cause a drop in autophagy markers (Ding et al., 2013). Grootaert et al. (2018) advocate that autophagy coordinates a “fight (autophagy), adapt (senescence) or die (apoptosis/necrosis)” mechanism in plaque VSMCs. According to this model, VSMCs initially “fight” the stressful insults posed by oxidized lipids, by boosting autophagy to promote their removal (Hill et al., 2008; Ding et al., 2013). In this manner, functional autophagy promotes plaque stability by modulating a “phenotypic switch” in VSMCs from a contractile to a synthetic phenotype (essential for ECM production) and inhibits cell death (Salabei and Hill, 2013). However, when the cells’ “fighting” resources are exhausted, they initiate an “adaptation” response by undergoing proliferative arrest, with an associated increase in senescence markers and eventually cell death, contributing to plaque instability (Grootaert et al., 2015, 2018). We refer to two excellent reviews for more details on the role of autophagy in atherosclerosis (Evans et al., 2017; Grootaert et al., 2018). For an overview of the relationship between autophagy-lysosomal homeostasis and cellular senescence in atherosclerosis please refer to our recent review (Machado-Oliveira et al., 2020).

### Efferocytosis

Efferocytosis is a multistep process of engulfment of dead cells by phagocytes that allows multicellular organisms to recycle cellular components (Boada-Romero et al., 2020). The removal of cellular corpses is important in both homeostasis and disease. In advanced atherosclerosis, the death of macrophages coupled with defective phagocytic clearance of foam cells, promotes secondary necrosis, plaque expansion and rupture, ultimately leading to acute coronary syndromes and stroke (Poon et al., 2014; Boada-Romero et al., 2020). Monocyte-derived macrophages are initially recruited into the developing lesions by “find-me” chemotactic factors, such as chemokines and lipids, secreted by dying cells (Yurdagul et al., 2018). At this stage, macrophages display rapid and efficient efferocytosis, effectively preventing the leakage of immunogenic peptides and lipid contents from the apoptotic cells (Nagata et al., 2010; Figure 1C). This restricts plaque progression by tempering post-apoptotic necrosis and inflammation (Henson et al., 2001). Engulfment of apoptotic cells by macrophages is made possible by an array of receptors that bind, directly or indirectly, to “eat-me” signals displayed on the surface of the dying cells. Live cells, in turn, are protected by the presence of “don’t eat-me” signals on their surface (Yurdagul et al., 2018). Upon recognition, the internalization of apoptotic cells results in the formation of the so-called phagocytic cup, which then proceeds with the retraction of the phagosome into the cell (Yurdagul et al., 2018). After this step, the phagosome can follow one of two routes, both heavily reliant on the lysosome-autophagy axis. On the canonical route, phagosome maturation is mediated through the activity of VPS34, phosphatidylinositol-3-phosphate (PI3P), Rab proteins and other molecular traffic machinery (Vieira et al., 2001; Flannagan et al., 2012). The fusion of the late phagosome with lysosomes allows lysosomal hydrolases to degrade the phagocytic cargo. Maturation of the phagocyte is accompanied by increasing acidification in the lumen, mediated by the v-ATPase (Martinez, 2017). Alternatively, the phagosome can be modified to either facilitate or impair its maturation. One such modification occurs during LC3-associated phagocytosis (LAP), the second possible route, characterized by the recruitment of LC3 to the phagosomal membrane (Martinez et al., 2011; Santarino et al., 2017). This process is carried out by a subset of proteins required for canonical autophagy (ATG proteins) in combination with unique LAP regulators (Martinez et al., 2015). LAP promotes the rapid maturation of the phagosome, and lysosomal fusion. After degradation of the apoptotic cells in phagolysosomes, macrophages become overloaded with macromolecular constituents and need therefore to either consume or efflux this cargo (A-Gonzalez et al., 2009; Martinez, 2017).

As discussed earlier, autophagy, if not excessive, protects macrophages against cell death. This is illustrated by the fact that blocking autophagy worsens the recognition and clearance of dead cells by efferocytes and promotes plaque necrosis (Liao et al., 2012). Presently it is unclear why efferocytosis fails in advanced atherosclerosis. Most studies propose a dysregulation of “eat-me” and “don’t eat-me” signals (Kojima et al., 2016; Cai et al., 2017). However, we and others (Sergin et al., 2015) suggest a central role for lysosomal dysfunction in this process. Although a direct link between progressive lysosomal dysfunction and compromised efferocytosis has not been investigated, impaired lysosomal acidification and reduced hydrolase activity can be postulated to adversely impact the ability of macrophages to handle exogenous phagocytic cargo. Substantiating this view, studies have proved the existence of a link between lysosomal storage and impaired phagocytosis (Berg et al., 2016). Berg et al. (2016) show that impaired lysosomal protease activity and consequent accumulation of undigested material in macrophages, disrupt the endocytic recycling and impair migration to, and thus engulfment of, dying cells. In advanced plaques, unengulfed apoptotic macrophages also start to accumulate and undergo secondary necrosis. Another recent publication links the inhibition of LAL with defective oxysterol production, which in turn causes inflammasome activation, decreased cholesterol efflux and impaired apoptotic cell clearance by macrophages (Viaud et al., 2018).

## Treating Atherosclerosis by Targeting Lysosomes—The Lessons to Be Learned From LSDs

Scientists in the LSD field have been developing strategies to circumvent the defects in lysosomal function associated with the pathologies. These pharmacological and genetic strategies may aim to directly correct the protein defect, to ameliorate pernicious secondary effects or to provide a general boost in lysosome function (Platt, 2018; Marques and Saftig, 2019). Despite recent positive outcomes in clinical trials with therapeutics for the treatment of atherosclerosis, namely those based on monoclonal antibodies against IL-1β and PCSK9 (Ridker et al., 2017; Sabatine et al., 2017), this disease remains the leading cause of death worldwide. Targeting lysosomal dysfunction by making use of the numerous tools developed by the LSD field may have a positive impact on this ongoing search for improved therapeutics against atherosclerosis. The studies discussed in this section are listed in Supplementary Table 1.

### LAL Supplementation

**FIGURE 2:**
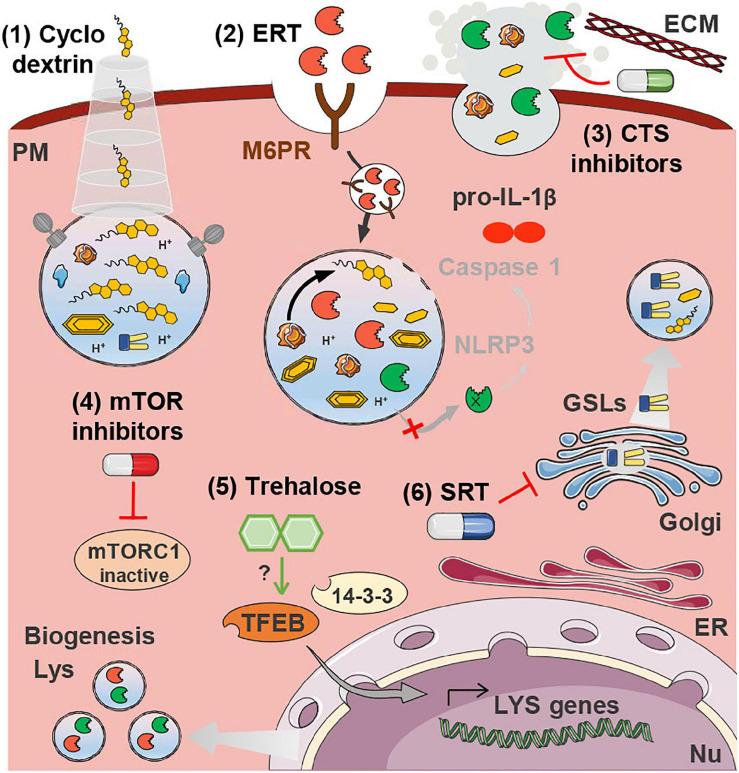
Therapeutics for atherosclerosis targeting lysosomal dysfunction. Cyclodextrin **(1)** releases the FC sequestered inside lysosomes. ERT **(2)** is based on the delivery of recombinant LAL that is taken up by mannose-6-phosphate receptors (M6PR) and directed to the lysosomes. In the acidic lumen of lysosomes LAL is released from the M6PR and catalyzes the hydrolysis of accumulated CE. Cathepsin (CTS) inhibitors **(3)** may be beneficial to plaque stability by preventing the degradation of the ECM, and by decreasing the aggregation of LDL, which hampers its uptake by non-regulated SRs. Both mTOR inhibitors **(4)** and trehalose **(5)** drive lysosome biogenesis by stimulating the translocation of TFEB to the nucleus where it drives the transcription of lysosomal genes. SRT **(6)** is based on the inhibition of the (glucosylceramide) synthase responsible for the production of glycosphingolipids (GSLs) that would otherwise accumulate inside lysosomes. All these strategies, with the exception of (3), have the potential to decrease the accumulation of lysosomal substrates and thereby restore membrane integrity, preventing cathepsin release and NRLP3 inflammasome activation (IL-1β production and release). This has only been experimentally demonstrated in the case of trehalose treatment. Some images in this figure were adapted with permission from Servier Medical Art, licensed under a Creative Common Attribution 3.0 Generic License (http://smart.servier.com/).

There is currently no curative treatment for LSDs. Although gene therapy has made significant strides toward this goal in the last years, the standard of care for most LSDs (particularly the non-neuronopathic forms) is still enzyme replacement therapy (ERT). ERT consists in the supplementation with exogenous recombinantly produced lysosomal hydrolases delivered intravenously (and more recently also intracranially) (Platt, 2018; Schulz et al., 2018). ERT with recombinant LAL (sebelipase alfa) is the standard therapy for Wolman disease (LAL deficiency) and CESD since 2015 (Burton et al., 2015; Wilson et al., 2018). LAL plays a crucial role in the hydrolysis of lipoprotein-derived CE and TG during atherogenesis. As already mentioned, LAL inhibition in macrophages decreases the export of cholesterol via ABCA1 and impairs oxysterol production and the efferocytosis of dead cells (Viaud et al., 2018). Additionally, the gene encoding for LAL (LIPA) was identified as a susceptibility gene for coronary artery disease by several genome-wide association studies (Peden et al., 2011; Wild et al., 2011). Given the central role of LAL in the etiology of atherosclerosis, scientists have hypothesized that supplementation with recombinant enzyme might also represent a valid strategy to halt disease progression (Du and Grabowski, 2004; Dubland and Francis, 2015; Figure 2). LAL augmentation was able to decrease atherosclerosis in a murine model (LDLR knock-out mice) (Du et al., 2004) and the use of recombinant LAL for the treatment of atherosclerosis continues to be investigated (Grabowski and Du, n.d.). On the downside, ERT strategies present substantial limitations regarding the elevated production costs of recombinant enzymes, adverse immunological reactions and the invasiveness of the delivery methods (Marques and Saftig, 2019). There is also a knowledge gap concerning the effects of systemic delivery of a recombinant hydrolase to individuals not carrying a genetic deficiency. Nanotechnology could prove a valuable asset for overcoming these limitations, by allowing local and controlled delivery of the therapeutic enzyme (Del Grosso et al., 2019; Kim et al., 2020a). Nonetheless, even under these circumstances, augmenting the hydrolysis of lipoprotein CE may increase lysosomal membrane FC and thereby exacerbate lysosomal dysfunction. Further studies are thus warranted to firmly establish the safety of this approach.

### Substrate Reduction Therapy

Substrate reduction therapy (SRT) is an alternative therapeutic approach often employed in LSDs whenever treatment by ERT is not feasible. Approved SRT strategies involve the pharmacological inhibition of the synthesis of glycosphingolipids (GSLs), a class of membrane lipids that undergo lysosomal breakdown and that is often a primary or secondary storage product of LSDs (Aerts et al., 2017; Platt, 2018). Multiple GSLs, such as the building block ceramide, are bioactive lipids, and impairment of their degradation may thus have considerable consequences for pathological processes. Similarly to LSDs, accumulation of GSLs has been demonstrated in human and murine atherosclerotic lesions (Garner et al., 2002), as well as in the circulation of atherosclerosis patients (Dawson et al., 1976; Chatterjee et al., 1997). The accumulation of these metabolites is most likely a consequence of the general impairment of lysosomal degradative capacity. Many of these storage lipids have been associated with pro-atherogenic properties. For example, ceramide increases lipoprotein aggregation (Park et al., 2006) and lactosylceramide promotes cholesterol accumulation (Gong et al., 2004). GSLs in general have been associated with inflammation and plaque instability (Park et al., 2006; Edsfeldt et al., 2016). Accordingly, several groups have demonstrated that pharmacological inhibition of GSL synthesis, through the inhibition of the enzyme glucosylceramide synthase (the enzyme that catalyzes the synthesis of the precursor of complex GSLs) with different drugs, ameliorates atherosclerosis in mouse models (Bietrix et al., 2010; Chatterjee et al., 2014; Mishra et al., 2015; Yu et al., 2019; Figure 2). The use of the serine palmitoyltransferase inhibitor myriocin, blocking the first step in sphingolipid biosynthesis, also improved atherosclerosis (Park et al., 2004; Hojjati et al., 2005). One study, however, found no atheroprotective effects concomitant to the reduction of GSLs in atherosclerosis-prone ApoE-deficient mice (Glaros et al., 2008). Further studies are thus required in order to confirm the involvement of GSLs in the atherogenic cascade as well as their potential as therapeutic targets for atherosclerosis.

### Cyclodextrins

The events taking place locally in the arterial wall share a lot of similarities with the events occurring systemically in NPC patients and animal models. In NPC disease, the macrophages are also particularly affected by the intralysosomal FC accumulation due to defective export machinery. The NPC1 protein has been shown to protect LDLR-deficient mice against the development of atherosclerosis through the regulation of LXR-dependent cholesterol efflux and mitigation of cholesterol-induced oxidative stress in macrophages (Welch et al., 2007; Zhang et al., 2008). It is therefore not surprising that therapies with demonstrated potential to treat NPC disease are also being investigated in the context of atherosclerosis, the clinical use of cyclodextrins being a prime example. Cyclodextrin (2-hydroxypropyl-β-cyclodextrin) was initially devised as a vector to deliver the neurosteroid allopregnanolone to an NPC mouse model. However, pre-clinical trials revealed the positive effects in animal longevity to be caused by the cyclodextrin-mediated release of the cholesterol sequestered in the late endosome-lysosome pool into the cytosolic pool (Griffin et al., 2004; Davidson et al., 2009; Figure 1E). While clinical trials for the treatment of NPC with cyclodextrin are still ongoing (Ory et al., 2017; Hastings et al., 2019), they have in the meantime triggered the interest of the CVD research field. Pre-clinical studies have shown that cyclodextrin can promote atherosclerosis regression in ApoE^–/–^ mice by reprogramming macrophages (i.e., increased oxysterol production and consequent LXR-mediated increase of cholesterol efflux) (Zimmer et al., 2016). Additionally, VSMCs and ECs treated with different cyclodextrins showed reduced cellular cholesterol content and modulated expression of ABC transporters involved in reverse cholesterol transport (Coisne et al., 2016). Despite the potential health benefits, the use of cyclodextrin has been linked to significant hearing loss in animal models and humans (Crumling et al., 2017). New formulations of cyclodextrin continue to be investigated that could assist in the mitigation of this pernicious side-effect (Kim et al., 2020b, c).

### mTOR Inhibitors

Recent studies have demonstrated that mTOR inhibitors have unique anti-atherosclerotic effects, including induction of autophagy, depletion of plaque macrophages, activation of cholesterol efflux and inhibition of pro-inflammatory signaling (Castro et al., 2004; Pakala et al., 2005; Mueller et al., 2008; Kurdi et al., 2016; Zhang K. S. et al., 2019; Figure 1E). However, a major shortcoming of this approach is the associated dyslipidemia (Holdaas et al., 2015). Several strategies, such as combination therapy with statins and metformin, have been suggested to oppose adverse side-effects caused by mTOR inhibitors. Statins and metformin are two drugs widely used to reduce cardiovascular risk that are also known to inhibit mTORC1 indirectly via 5′ adenosine monophosphate-activated protein kinase (AMPK) activation (Sun et al., 2006; Seneviratne et al., 2020). These approved drugs may thus be ideal candidates for exploiting the full potential of mTORC1 inhibition in the treatment of atherosclerosis (Martinet et al., 2014; Kurdi et al., 2017). This strategy may also potentiate the correct polarization of macrophages via the inhibition of the PI3K/Akt/mTOR signaling pathway (Sun et al., 2018).

### Cathepsin Modulators

Lysosomal cathepsins, particularly when present in the extracellular milieu, have been implicated in the etiology of CVDs (Cheng et al., 2011; Liu et al., 2018). The extra-lysosomal activity of these proteases mediates several atherogenic processes, such as immune and vascular cell recruitment, angiogenesis, oxLDL degradation, ECM remodeling and cell death (Cheng et al., 2011; Liu et al., 2018). The importance of these proteases for the atherogenic cascade has been demonstrated by numerous studies evidencing that their genetic or pharmacological ablation (namely of cathepsin C, K, L, and S) hinders atherosclerotic plaque formation (Sukhova et al., 2003; Bengtsson et al., 2005; Lutgens et al., 2006; Kitamoto et al., 2007; Figueiredo et al., 2015; Herías et al., 2015). Altogether these studies suggest that, besides being potential biomarkers for CVDs, cathepsin proteases may also represent promising therapeutic targets to delay plaque progression (Figure 2). Given the crucial role of these enzymes in cell homeostasis, therapeutic approaches targeting them must find a delicate balance between inhibition of excessive extracellular activity and maintenance of their physiological functions.

### Boosting Lysosome Biogenesis

TFEB is the master regulator of autophagy and lysosome biogenesis (Sardiello et al., 2009). Consequently, overexpression of this transcription factor increases the number of lysosomes and enhances lysosomal catabolic activity. This feature could be crucial in a pathophysiological context in which old lysosomes become dysfunctional and undegraded cargo accumulates (Sardiello et al., 2009). Scientists in the field of atherosclerosis were motivated by these observations to try to ameliorate atherosclerosis and vascular aging through the pharmacological and genetic enhancing of the macrophage autophagy-lysosomal system (LaRocca et al., 2013; Emanuel et al., 2014). *In vitro* studies have demonstrated that increasing lysosomal biogenesis through TFEB overexpression can rescue the lipid-induced lysosomal dysfunction, reduce inflammasome activation and decrease atherosclerosis progression (Emanuel et al., 2014). These findings were confirmed by *in vivo* studies reporting the reduction of atherosclerosis in murine models through the overexpression of macrophage TFEB (Sergin et al., 2017). Boosting lysosome biogenesis in mouse macrophages reversed the observed defects in autophagy by enhancing their degradative capacity, stimulating the selective degradation of p62-enriched protein aggregates (aggrephagy) and halting macrophage apoptosis and pro-inflammatory IL-1β levels (Sergin et al., 2017; Evans et al., 2018). Clearance of lipid-laden lysosomes by exocytosis may also represent a TFEB-dependent mechanism for the alleviation of the autophagy-lysosomal system (Medina et al., 2011). Moreover, boosting TFEB-mediated lysosome clearance was shown to be beneficial to cardiac cells (Ma et al., 2012; Pan et al., 2017). In atherosclerotic plaques, stimulating exocytosis may, however, not be entirely beneficial, as it could contribute to the release of partially degraded and oxidized lipoproteins to the extracellular space, promoting atherogenesis.

Notably, the group of Razani has also demonstrated the feasibility of employing the non-reducing natural disaccharide trehalose as a means of pharmacologically boosting lysosome biogenesis and recapitulating the atheroprotective effects of TFEB overexpression (Sergin et al., 2017; Figure 2). Previous studies by others had already shown that pharmacological induction of autophagy with the polyamine spermidine could reverse arterial aging (LaRocca et al., 2013). Trehalose is also a potent inducer of autophagy, through yet unknown mechanisms, and is able thereby to ameliorate various protein aggregation neurodegenerative diseases (Tanaka et al., 2004; Sarkar et al., 2007; Rodríguez-Navarro et al., 2010). Trehalose also induces the translocation of the MiT/TFE family member TFE3, but not of MITF (Sergin et al., 2017). TFE3 and TFEB regulate overlapping sets of genes and their overexpression has similar effects (Martina et al., 2014). These two transcription factors demonstrate a cooperative and partially redundant effect and function as master integrators of different stress pathways, controlling the expression of key genes involved in the modulation of the immune response, metabolism, mitochondrial homeostasis, and unfolded protein response (Martina et al., 2014; Pastore et al., 2016; Martina and Puertollano, 2017). The individual importance of each transcription factor for plaque cells under pro-atherogenic conditions remains an open question.

A potential drawback of boosting MiT/TFE expression could be the unwanted deregulation of cell proliferation control (Marques and Saftig, 2019). Underlying this concern is the fact that MiT/TFE proteins have demonstrated oncogenic features in multiple malignancies, including melanoma and renal cell carcinoma (Haq and Fisher, 2011; Kauffman et al., 2014; Perera et al., 2019). There are thus well-founded concerns that excessive MiT/TFE activity could promote malignancy development, namely through an hyperactivation of the WNT pathway (Calcagnì et al., 2016). Future research must ascertain the safety of these therapeutic strategies, perhaps by focusing on local and transient ways of boosting lysosome biogenesis.

#### Alternative Regulators of Lysosome Biogenesis

TFEB-mediated induction of lysosome biogenesis does not paint a complete picture of the intricate regulation of the autophagy-lysosomal system. The activation of a pro-degradative transcriptional program can involve other molecular players. Among the transcription factors shown to modulate the expression of lysosomal and autophagy genes can be counted the FoxO transcription factors, the signal transducer and activator of transcription 3 (STAT3) and c-MYC (Kreuzaler et al., 2011; Shin et al., 2016; Yan et al., 2016; Martínez-Fábregas et al., 2018; Annunziata et al., 2019). The unique feature of these three transcription factors is that they have also been reported to influence the development of atherosclerosis (Napoli et al., 2002; Tsuchiya et al., 2012; Wang et al., 2016; Yang X. et al., 2020). However, it remains unclear whether this involvement is related to their role in the homeostasis of the lysosome-autophagy axis.

## Conclusion

Almost 50 years after its publication, the original observation by De Duve, the scientific “father” of lysosomes, has been vindicated. A large body of evidence has been produced confirming the central role played by lysosomal dysfunction in atherogenesis. Such studies have demonstrated that lysosomes alone must process the lipoproteins trapped in the arterial wall during atherosclerotic plaque development. While their catalytic function in ECs, VSMCs and macrophages—the main cell types of the arterial wall—remains intact in the initial stages of plaque development, eventually the influx of substrates surpasses the capacity of these small organelles. The advent of extracellular and/or intracellular modifications in the lipoproteins, throws a final wrench into the endolysosomal compartment, halting its primary function as the degradative compartment of the cell and, as a consequence, its secondary (albeit crucial) functions.

Although significant progress has been made in the understanding of the etiology and the consequences of lysosomal dysfunction in arterial plaque cells, substantial questions remain. Scientists are still to clarify, among other questions: exactly where and how modifications in lipoproteins occur; which components of oxLDL underlie its atherogenic properties; and how the different transcription factors orchestrate the response to a dysfunctional lysosomal compartment. Here we argue that, in order to answer these questions, scientists in the field may benefit from the knowledge gathered since the 1950’s by researchers studying LSDs, monogenic inherited disorders affecting the functioning of lysosomes. The advantages of the cooperation between these domains of expertise are already evident. For example, initial observations by scientists investigating LSDs enabled researchers to study the key role of the transcription factor TFEB, the master regulator of lysosome biogenesis, in the development of atherosclerosis. Additionally, many markers of autophagy-lysosomal dysfunction may also be employed as (bio)markers for atherosclerosis prognosis (e.g., p62 aggregates, extracellular LAL levels, circulating cathepsin levels, etc.). Finally, this cooperation has already brought to light the fact that therapeutic interventions aiming to compensate the individual deficiencies in lysosomal proteins or to boost the autophagy-lysosomal machinery as a whole, hold considerable potential for the treatment of atherosclerosis. The knowledge thus gathered may assist in the ongoing efforts to minimize the morbidity and mortality associated to atherosclerosis.

## Author Contributions

OV proposed and structured the outline of the manuscript. CR did the initial literature research. AM wrote the majority of the manuscript and designed the figures. All authors have contributed to the writing and reviewed the manuscript, read and agreed to the published version of the manuscript.

## Conflict of Interest

The authors declare that the research was conducted in the absence of any commercial or financial relationships that could be construed as a potential conflict of interest.
