# Supplementary material for: Lysosome (Dys)function in Atherosclerosis—A Big Weight on the Shoulders of a Small Organelle
Source: Front Cell Dev Biol. 2021 Mar 29;9:658995. doi: 10.3389/fcell.2021.658995 (PMC8039146; doi:10.3389/fcell.2021.658995)
Supplement: Supplementary file 1 [file Data_Sheet_1.PDF]

Supplementary Table 1 – Summary of the experimental studies discussed in section 6. Selection of the main studies investigating the impact of the different therapeutic strategies targeting lysosomal dysfunction in atherosclerosis.

Therapeutic approach	Studies in atherosclerosis (selection)
ERT – LAL supplementation	(Du et al., 2004)<br>(Peden et al., 2011)<br>(Wild et al., 2011)<br>(Viaud et al., 2018)
SRT– Inhibition of GSL synthesis	(Park et al., 2004)<br>(Hojjati et al., 2005)<br>(Glaros et al., 2008)<br>(Bietrix et al., 2010)<br>(Chatterjee et al., 2014)<br>(Mishra et al., 2015)
Cyclodextrin	(Zimmer et al., 2016)<br>(Coisne et al., 2016)<br>(Kim, Han, et al., 2020)<br>(Kim, Kim, et al., 2020)
mTOR inhibitors	(Castro et al., 2004)<br>(Pakala et al., 2005)<br>(Mueller et al., 2008)<br>(Sun et al., 2018)<br>(Seneviratne et al., 2020)
Cathepsin modulators	(Sukhova et al., 2003)<br>(Bengtsson et al., 2005)<br>(Lutgens et al., 2006)<br>(Kitamoto et al., 2007)<br>(Herías et al., 2015)<br>(Figueiredo et al., 2015)
Boosting lysosome biogenesis	(Razani et al., 2012)<br>(LaRocca et al., 2013)<br>(Emanuel et al., 2014)<br>(Sergin et al., 2017)
Alternative regulators of lysosomal biogenesis	(Napoli et al., 2002)<br>(Tsuchiya et al., 2012)<br>(Wang et al., 2016)<br>(Yang et al., 2020)
